# Incidence of acute myocardial injury and its association with left and right ventricular systolic dysfunction in critically ill COVID-19 patients

**DOI:** 10.1186/s13613-022-01030-8

**Published:** 2022-06-21

**Authors:** Saga Jansson, Patrik Johansson Blixt, Helen Didriksson, Carina Jonsson, Henrik Andersson, Cassandra Hedström, Jan Engvall, Meriam Åstrom Aneq, Michelle S. Chew

**Affiliations:** 1 Department of Anaesthesiology and Intensive Care, Biomedical and Clinical Sciences, Linköping University, Linköping, Sweden; 2 Department of Clinical Physiology and Department of Health, Medicine and Caring Sciences, Linköping University, Linköping, Sweden

**Keywords:** COVID-19, Intensive care, Acute myocardial injury, Ventricular dysfunction, Echocardiography, Cardiac troponins

## Abstract

**Background:**

Previous studies have found an increase in cardiac troponins (cTns) and echocardiographic abnormalities in patients with COVID-19 and reported their association with poor clinical outcomes. Whether acute injury occurs during the course of critical care and if it is associated with cardiac function is unknown.

The purpose of this study was to document the incidence of acute myocardial injury (AMInj) and echocardiographically defined left ventricular (LV) and right ventricular (RV) systolic dysfunction in consecutive patients admitted to an intensive care unit (ICU) for COVID-19. The relationship between AMInj and echocardiographic abnormalities during the first 14 days of ICU admission was studied. Finally, the association between echocardiographic findings, AMInj and clinical outcome was evaluated.

**Methods:**

Seventy-four consecutive patients (≥18 years) admitted to the ICU at Linköping University Hospital between 19 Mar 2020 and 31 Dec 2020 for COVID-19 were included. High-sensitivity troponin-T (hsTnT) was measured daily for up to 14 days. Transthoracic echocardiography was conducted within 72 h of ICU admission. Acute myocardial injury was defined as an increased hsTnT > 14ng/l and a > 20% absolute change with or without ischaemic symptoms. LV and RV systolic dysfunction was defined as at least 2 abnormal indicators of systolic function specified by consensus guidelines.

**Results:**

Increased hsTnT was observed in 59% of patients at ICU admission, and 82% developed AMInj with peak levels at 8 (3–13) days after ICU admission. AMInj was not statistically significantly associated with 30-day mortality but was associated with an increased duration of invasive mechanical ventilation (10 (3–13) vs. 5 days (0–9), *p*=0.001) as well as ICU length of stay (LOS) (19.5 (11–28) vs. 7 days (5–13), *p*=0.015). After adjustment for SAPS-3 and admission SOFA score, the effect of AMInj was significant only for the duration of mechanical ventilation (*p*=0.030).

The incidence of LV and RV dysfunction was 28% and 22%, respectively. Only indices of LV and RV longitudinal contractility (mitral and tricuspid annular plane systolic excursion) were associated with AMInj. Echocardiographic parameters were not associated with clinical outcome.

**Conclusions:**

Myocardial injury is common in critically ill patients with COVID-19, with AMInj developing in more than 80% after ICU admission. In contrast, LV and RV dysfunction occurred in approximately one-quarter of patients. AMInj was associated with an increased need for mechanical ventilation and ICU LOS but neither AMInj nor ventricular dysfunction was significantly associated with mortality.

**Supplementary Information:**

The online version contains supplementary material available at 10.1186/s13613-022-01030-8.

## Background

Severe Acute Respiratory Syndrome Coronavirus 2 (SARS-CoV-2) causes a plethora of symptoms and complications, affecting several organ systems, including the cardiovascular system [1]. The mechanisms behind how COVID-19 harms the heart remain unclear, and include direct damage to cardiomyocytes, an exaggerated immune response causing indirect damage, hypoxia, coronary spasm, microthrombi and/or direct endothelial/vascular injury, and potential injury from cardiotoxic drugs [2, 3]. The prevalence of myocardial injury in COVID-19 ranges from 7 to 62.6% [1]. This wide range may be explained by different patient populations, definitions, and the assays used in underlying studies. In addition, it may not reflect the entire population, since cardiac troponins (cTns) are often not routinely analyzed.

Myocardial injury appears to be linked to severity of COVID-19 disease and mortality [4–10]. Mortality from COVID-19 is reported to be higher among hospitalized patients with myocardial injury, compared to those without, [4, 6, 7, 11, 12]. Indicating that myocardial injury may portend a poorer prognosis. However, since severe hypoxaemia, cardiovascular and other comorbidities are common among hospitalized COVID-19 patients, it is conceivable that preexisting myocardial injury exists in a proportion of patients. It is not known if critical illness itself is an antecedent event for further myocardial injury, and if the occurrence of an acute injury during intensive care may be associated with adverse outcomes. Although many studies report on the incidence of myocardial injury, none have explored whether myocardial injury is an acute event. The incidence of acute myocardial injury (AMInj) according to current consensus definitions [13] requires a demonstrable dynamic change in cTns. This may be clinically important as treatment and prognosis of these patients may differ to patients with increased levels of cTn but without any acute injury. The association between AMInj and cardiac function abnormalities has not been well investigated.

Studies show that left ventricular (LV) and right ventricular (RV) abnormalities occur commonly in hospitalized COVID-19 patients [17, 18]. Cardiac function is most often measured using echocardiography in the critically ill. RV abnormalities appear to be more common than LV abnormalities and suggest an association between RV dysfunction, disease severity and mortality [19, 20]. Yet, a comprehensive review of echocardiographic studies of COVID-19 patients reported preserved global LV function, and variable findings regarding the RV. Specifically, normal echocardiographic findings were found in about 50% of subjects, with usually unaffected left ventricular ejection fraction. Although RV dysfunction seemed more likely associated with increased mortality, insufficient information was available to draw robust conclusions about this relationship [21].

Thus, several important knowledge gaps remain. First, only few studies exist for unselected critically ill patients with systematic data collection. Therefore, the true incidence of echocardiographically defined ventricular dysfunction is not known. Second, we are not aware of any studies investigating the incidence of AMInj defined by consensus guidelines among critically ill patients admitted to ICUs for COVID-19 disease. Third, it is unknown whether left and right ventricular dysfunctions are related to AMInj in critically ill COVID-19 patients.

The aim of this study was to investigate the incidence of AMInj the first 14 days of ICU admission and echocardiographically defined left and right ventricular systolic dysfunctions during the first 72 h of ICU admission in consecutive patients admitted to the intensive care unit at a tertiary hospital in Sweden. Furthermore, we investigated the relationship between AMInj and echocardiographic abnormalities. A secondary aim was to evaluate the association between echocardiographic findings, AMInj and clinical outcome.

## Methods

This is a retrospective, cohort study of patients with confirmed SARS-CoV-2 infection and COVID-19 disease admitted to the ICU of Linköping University Hospital in Sweden during the 2020 pandemic. The study was approved by the Swedish Ethical Review Authority (Dnr 2020-01884) without the requirement for written informed consent from individual patients due to its retrospective, observational nature.

### Patients

Adult patients ≥18 years of age admitted to the ICU at Linköping University Hospital with COVID-19 from 1 March to 31 December 2020 were identified with the ICD-10 diagnosis code U07.1 (COVID-19, virus identified), with SARS-CoV2 confirmed by polymerase chain reaction.

### Data extraction

A blinded observer not involved in the care of the patients extracted data using a predefined template. Baseline characteristics including comorbidities, intensive care treatment, laboratory and echocardiographic variables and outcomes were registered.

Biomarker data were documented for the first 14 days of ICU admission, or until discharge. Plasma samples and clinical parameters were collected at 0600 in all ICU patients according to departmental routine. Elevated NT-proBNP was defined as > 150 ng/l (< 60 years) or > 300 ng/l (≥60 years). AMInj was defined as an increased hsTnT > 14ng/l and a > 20% absolute change with or without ischaemic symptoms [13].

Transthoracic echocardiography was performed within 72 h of ICU admission in all patients according to departmental routine. A GE Vingmed Ultrasound Vivid E95 or Vivid S70 echocardiography scanner with a 1.5–4.5 MHz (M5S-D) transducer (GE Healthcare GmbH, Solingen, Germany) was used for data acquisition. An echocardiogram was performed according to a prespecified COVID-19 protocol by experienced sonographers or a clinical physiologist (>1000-h echocardiography experience). All imaging was conducted in the ICU using a COVID-19 dedicated scanner with standard operating protocols for personal protective equipment and cleaning. Images were transferred to ViewPoint (v. 6.10.1, GE Healthcare GmbH, Solingen, Germany) with EchoPAC Suite (GE EchoPAC PC Software v. 202, GE Vingmed Ultrasound AS, Horten, Norway). At least three heart beats were recorded in each view for patients with sinus rhythm. We endeavoured to capture at least five beats in patients with atrial fibrillation. Recorded value for each variable is the average of these beats. Data were acquired without consideration for the phase of respiration and analysed by a blinded observer not involved in clinical care of patients. Left ventricular systolic dysfunction [22, 23] was defined as any 2 of the following: LVEF ≤ 50%, average mitral (lateral and medial) S’ by colour tissue Doppler velocity < 5 cm/s, MAPSE (average lateral and medial) <10 mm or GLS (average of apical 4-chamber and 2-chamber views) > −15%. Right ventricular systolic dysfunction [22, 23] was defined as any 2 of the following: Tricuspid Annular Plane Systolic Excursion (TAPSE) <17 mm, RV:LV End Diastolic Area (EDA) ratio >0.6, tricuspid Sʹ by colour tissue Doppler velocity <6 cm/s [22], Fractional Area Contraction (FAC) < 35%, Free Wall Strain (FWS) > −20%. A minimum of 2 criteria were chosen to decrease the possibility of false positives. We endeavoured to adhere to the PRICES statement for reporting echocardiography studies, a summary of this checklist is included in Additional file 1: Table S1 [23].

### Statistical considerations

Categorical variables are given as frequencies or percentages. Continuous variables are described using means and standard deviations, or alternatively medians and interquartile ranges, depending on the distribution. *T* tests, Mann–Whitney tests are used for comparison of continuous data, and chi-squared or Fisher exact tests for categorical and frequency data.

To explore different definitions of myocardial injury and to make our results comparable to previous studies, we also conducted sensitivity analyses using 2 other definitions: myocardial injury (hsTnT > 14 ng/l) and severe myocardial injury (hsTnT > 45 ng/l which is approximately 3× the 99th percentile of a normal healthy population, and in line with median values of previous studies in the critically ill and in COVID populations) [6, 24, 25]. Sample size was not prespecified as we aimed to include all available patients during the study period. Given the exploratory nature of the research, more emphasis is placed on the size of the effects found than on the outcome of significance tests (although these are performed with a critical *p *< 0.05 for each test). All echocardiographic, laboratory and clinical outcomes data were analyzed by an independent researcher not involved in clinical care.

## Results

**Fig. 1:**
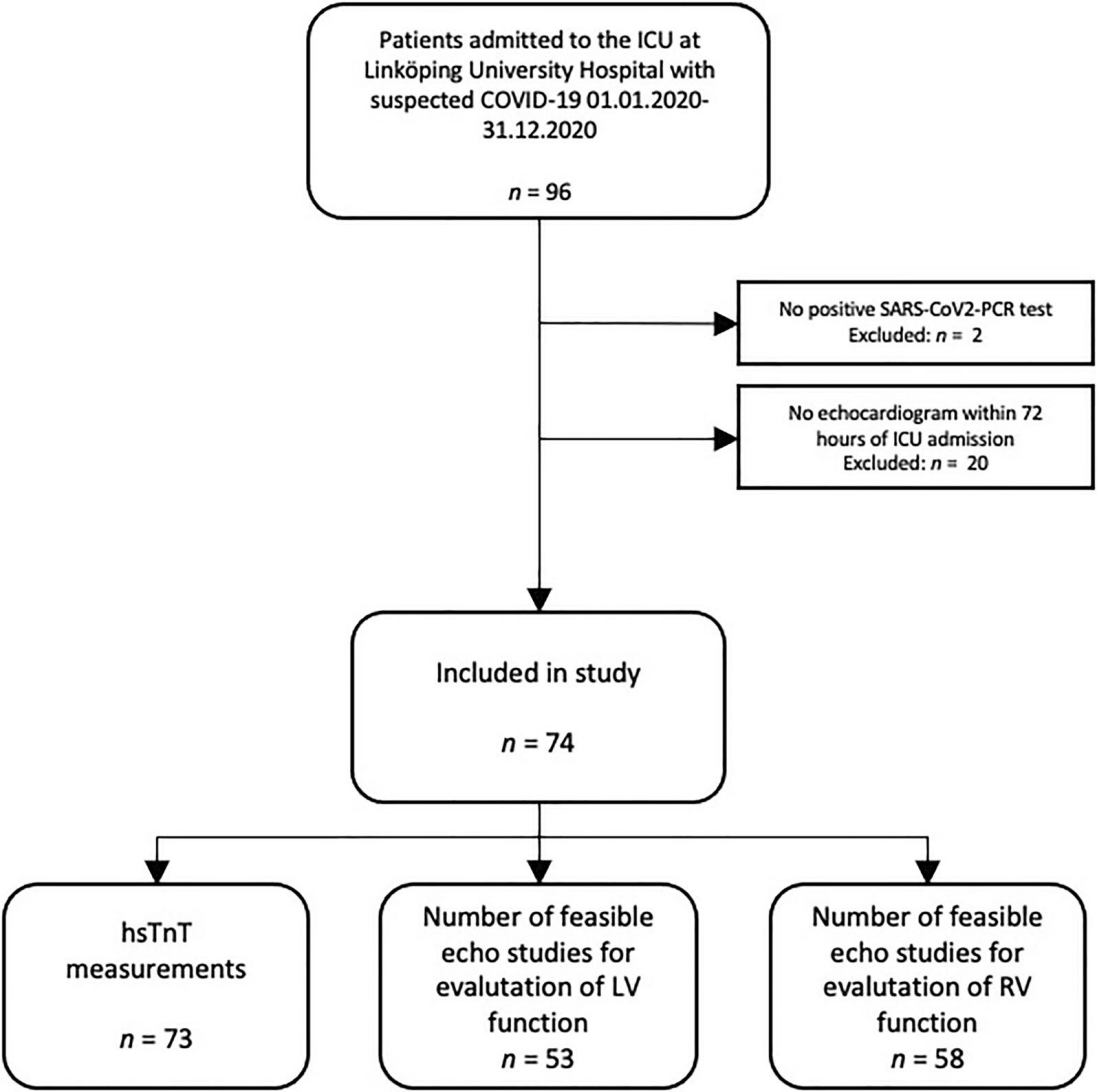
Inclusion flowchart

All patients admitted to the intensive care unit at Linköping University Hospital were screened for this study. A flow chart of inclusion and exclusion is shown in Fig. 1. A total of 74 patients were finally included in the study.

Baseline and clinical characteristics of the study population are shown in Table 1. A majority of patients (80%) suffered from comorbidities, of whom 57% had hypertension, 24% had diabetes mellitus and 11% had chronic renal disease, all known risk factors for cardiovascular disease. The proportion of patients with preexisting cardiac disease (arrythmias, heart failure or ischaemic heart disease, or any combination of these) was 24%.

**Table 1:** Baseline characteristics and biomarker findings in all patients and stratified by acute myocardial injury

	All patients *n = *74	No AMInj *n =* 13	AMInj *n = *60	*P* value
Age	62.5 (56–72)	60 (55–62)	65 (59–73)	0.030
Sex, male	50 (68%)	5 (39%)	44 (73%)	0.023
BMI, kg/m^2^	28.7 (25.6–33.5)	27.8 (25.4–35.7)	28.7 (26–32.9)	0.751
SAPS-3	54 (49–62)	53 (49–56)	54.5 (50–63.5)	0.239
SOFA on admission	6 (4–8)	4 (4–6)	7 (4–8)	0.077
SOFA max	10 (7.5–12)	6 (5–6.5)	10.5 (8–12)	<0.001
CFS	3 (2–3)	3 (3–3)	3 (2–3)	0.144
Hypertension	42 (57%)	4 (31%)	38 (63%)	0.031
Diabetes	18 (24%)	2 (15%)	16 (28%)	0.498
Preexisting cardiac disease^a^	18 (24%)	3 (23%)	15 (25%)	1
Chronic respiratory disease	14 (19%)	2 (15%)	12 (20%)	1
Chronic renal disease	8 (11%)	0 (0%)	8 (13%)	0.336
Vasopressor days	10 (5–21)	3 (0–5)	13 (7–23)	<0.001
VIS, μg/kg/min^b^	4.34 (1.94–7.31)	1.24 (0.38–5.13)	5.04 (1.97–7.6)	0.040
CRRT	14 (19%)	1 (8%)	13 (22%)	0.440
hsTnT max, ng/l	53 (21–127)	12 (10–14)	71 (38–140)	<0.001
Day of peak hsTnT	8 (3–13)	2 (1–5)	10 (3–13)	0.002
NT-proBNP max, ng/l	1960 (755–5618)	760 (210–960)	2665 (870–6830)	<0.001
Day of peak NT-proBNP	4 (2–9)	3 (1–5)	7 (2–10)	0.027
Lactate at admission	1.2 (0.9–1.7)	1.2 (0.9–1.6)	1.3 (0.9–1.7)	0.977
Proportion requiring IMV	69 (93%)	9 (69%)	59 (98%)	0.067
IMV days	14.5 (6.75–25.5)	5 (0–9)	17.5 (8.5–28)	0.001
ICU LOS	17 (8–27.5)	7 (5–13)	19.5 (10.5–28)	0.015
ICU mortality	14 (19%)	1 (8%)	13 (22%)	0.440
30-day mortality	13 (18%)	0 (0%)	13 (22%)	0.107

Acute myocardial injury (AMInj) is defined as an increased hsTnT > 14ng/L and a > 20% absolute change with or without ischaemic symptoms [1]

Data are given as n (%) or median (IQR) unless otherwise indicated

*P* values refer to No acute myocardial injury vs. acute myocardial injury

*SAPS* Simplified Acute Physiology Score, *SOFA* sequential organ failure assessment, *VI* Vasopressor inotrope score, *CRRT* continuous renal replacement therapy, *hsTnT* high sensitivity Troponin T, *NT-proBNP* N-terminal pro B-type natriuretic peptide, *ICU* intensive care unit, *LOS* length of stay, *IMV* invasive mechanical ventilation

^a^Defined as arrythmia, heart failure or ischaemic heart disease

^b^Calculated as dopamine dose (μg/kg/min) + dobutamine dose (μg/kg/min) + 100x epinephrine dose (μg/kg/min) + 10x milrinone dose (μg/kg/min) + 10.000x vasopressin dose (U/kg/min) + 100x norepinephrine dose (μg/kg/min) [34]

### Acute myocardial injury during ICU admission

AMInj could be assessed in 73 of 74 patients, since one patient did not have at least 2 measurements during ICU admission. A majority of patients (60/73, 82%) suffered from acute myocardial injury according to the current consensus definition [13]. 82% had hsTnT above the 99th percentile of a healthy reference population during their ICU stay (i.e., myocardial injury, not necessarily acute), with 43 patients (59%) already increased at ICU admission.

Compared to patients without, those with acute myocardial injury were older, predominantly male, had a higher incidence of preexisting hypertension and had a higher peak NTproBNP (Table 1). Peak hsTnT occurred later in those with acute myocardial injury (day 10 (3–13) vs. day 2 (1–5) , *p*=0.002). Patients with acute myocardial injury required and spent more days on invasive mechanical ventilation, had higher maximal SOFA scores, higher vasopressor inotrope scores (VIS), were vasopressor/inotrope dependent for more days and stayed longer in the ICU (Table 1). The effect of AMInj regarding days on invasive mechanical ventilation was present even after adjustment for SAPS-3 and admission SOFA scores (Additional file 1: Table S2). Thirty-day mortality was 22% among patients with acute myocardial injury and 0% in patients without acute myocardial injury, a difference that did not reach statistical significance.

### Incidence of left and right ventricular systolic dysfunction using echocardiography

Although echocardiographic studies were available for all 74 patients, only 53 and 58 had sufficient data to confirm or refute a diagnosis of left or right ventricular dysfunctions, respectively, based on our criteria of a minimum of 2 positive variables. Fifteen of 53 (28%) of patients had echocardiographically defined LV dysfunction and 13 of 58 (22%) had RV dysfunction according our prespecified definitions. Fifteen percent had both LV and RV dysfunction (Tables 2 and 3). Of note, LV and RV GLS measurements were feasible in only 34 (46%) and 55 (74%) of patients, respectively. The most feasible parameters for LV systolic function were MAPSE (89%), VTI (86%) and s’ (84%). For RV systolic function, TAPSE (92%), FAC (93%) and RV:LV–EDA-ratio (92%) provided best feasibility. Respiratory settings, cardiovascular variables and use of vasopressors during echocardiography are provided in Additional file 1: Table S3.

**Table 2:** Echocardiography, biomarkers and clinical outcome in all patients and stratified by the presence of LV systolic function

	All patients	LV systolic function		*P* value
	*n = 74*	Normal *n* = 38	Abnormal *n* = 15	
Echocardiography *Left ventricle*				
LVEF, %	62 (53–67.5)	64 (59–69.5)	47 (39–55)	<0.001
LVEF < 50%	8 (13.6%)	0 (0%)	8 (53.5%)	<0.001
LV s’, cm/sec	6 (5–7)	6 (6–8)	4 (4–5)	<0.001
MAPSE, mm	11 (9–13)	11 (11–14)	7 (6–9)	<0.001
GLS	−17 (−21 to −15)	−20 (−22 to −17)	−13 (−14 to −10)	< 0.001
VTI, cm	18.7 (15.4 to 21.9)	19.4 (16.4 to 22.5)	17 (11.6 to 21.7)	0.177
*Right ventricle*				
TAPSE, mm	18 (16 to 22)	20.5 (18 to 23)	15 (12 to 17)	< 0.001
FAC, %	46 (39 to 53)	46 (41 to 54)	42 (33 to 48.5)	0.117
RVEDA:LVEDA	0.53 (0.46 to 0.57)	0.54 (0.46 to 0.57)	0.52 (0.45 to 0.57)	0.664
RV FWS	−23 (−27 to −21)	−25 (−29 to −21)	−23 (−25 to −16)	0.111
RV TV s’, cm/sec	10 (8 to 12)	11 (9 to 12)	7(6 to 9)	0.005
hsTnT max, ng/l	53 (21 to 127)	43 (16 to 105)	126 (50 to 284)	0.006
AMInj	60 (82%)	30 (78.9%)	14 (93.3%)	0.418
Vasopressor days	9.5 (5 to 21.25)	11.5 (6 to 23)	6 (4.5 to 13.5)	0.195
VIS, μg/kg/min	4.34 (1.94 to 7.31)	3.55 (1.5 to 6.69)	6.2 (3.64 to 7.73)	0.096
Proportion with IMV	69 (93%)	34 (90%)	14 (93%)	1
IMV days	14.5 (6.8 to 25.5)	15.5 (7 to 25)	16 (5 to 25.5)	0.737
SOFA max	10 (7.5 to 12)	9 (7 to 11)	10 (8 to 12)	0.304
ICU LOS, days	17 (8 to 27.5)	20 (11 to 32)	17 (6.5 to 26)	0.368
ICU mortality	14 (19%)	7 (18.4%)	2 (13.3%)	1
30-day mortality	13 (18%)	7 (18.4%)	2 (13.3%)	1

*P* values refer to normal vs. abnormal systolic function

*LVEF* left ventricular ejection fraction, *MV s’* mitral valve tissue colour doppler systolic velocity (average of septal and lateral measurements), *MAPSE* mitral annular plane systolic excursion, *GLS* global longitudinal strain, *VTI* velocity time integral measured at the left ventricular outflow tract, *TAPSE* tricuspid annular plane systolic excursion, *FAC* fractional area contraction, *EDA* end diastolic area, *FWS* free wall strain, *TV s’* tricuspid valve tissue colour doppler systolic velocity (free wall), *hsTnT* high sensitivity Troponin T, *VIS* vasopressor–inotrope score, *IMV* invasive mechanical ventilation. P values refer to normal vs. abnormal LV systolic function

**Table 3:** Echocardiography, biomarkers and clinical outcome in all patients and stratified by the presence of RV systolic function

	All patients	RV systolic function		*P* value
	*n* = 74	Normal *n* = 45	Abnormal *n* = 13	
Echocardiography *Left ventricle*				
LVEF, %	62 (53 to 67.5)	63 (57 to 70)	54 (50 to 63)	0.018
LVEF <50%	8 (13.6%)	4 (10.3%)	3 (25%)	0.334
LV s’, cm/sec	6 (5 to 7)	6 (4.9 to 6.93)	5 (4.77 to 7.41)	0.813
MAPSE, mm	10.75 (9 to 12.75)	11.25 (9.17 to 13.25)	8.88 (6 to 10.38)	0.004
GLS	−17 (−21 to −15)	−20 (−22 to −16)	−15 (−17 to −10)	0.022
VTI, cm	18.7 (15.4 to 21.9)	20.2 (15.7 to 23)	16 (12.3 to 17.9)	0.011
*Right ventricle*				
TAPSE, mm	18 (16 to 22)	20 (18 to 23)	15 (13 to 16)	<0.001
FAC, %	46 (39 to 53)	45 (41 to 54)	47 (34 to 48)	0.267
RVEDA:LVEDA	0.53 (0.46 to 0.57)	0.52 (0.46 to 0.57)	0.56 (0.53 to 0.64)	0.050
RV FWS	−23 (−27 to −21)	−25 (−30 to −22)	−18 (−23 to −13)	<0.001
RV TV s’, cm/sec	9.8 (8 to 12)	10 (8 to 12.3)	9 (6 to 9.4)	0.018
hsTnT max, ng/l	53 (21 to 127)	47 (16 to 117)	85 (52 to 239)	0.026
AMInj	60 (82%)	34 (75.6%)	13 (100%)	0.055
Vasopressor days	9.5 (5 to 21.25)	9 (4 to 19)	17 (6 to 24)	0.201
VIS, μg/kg/min	4.34 (1.94 to 7.31)	3.78 (1.31 to 6.94)	4.36 (1.05 to 7.6)	0.929
Proportion requiring IMV	69 (93%)	34 (90%)	14 (93%)	1
IMV days	14.5 (6.8 to 25.5)	14 (6 to 24)	20 (12 to 28)	0.204
SOFA max	10 (7.5 to 12)	9 (7 to 11.5)	9 (8 to 12)	0.503
ICU LOS	17 (8 to 27.5)	18 (8 to 28)	21 (15 to 27)	0.526
ICU mortality	14 (19%)	9 (20%)	1 (7.7%)	1
30-day mortality	13 (18%)	7 (15.6%)	2 (15.4%)	1

*P* values refer to normal vs. abnormal systolic function

*LVEF* left ventricular ejection fraction, *MV s’* mitral valve tissue colour doppler systolic velocity (average of septal and lateral measurements), *MAPSE* mitral annular plane systolic excursion, *GLS* global longitudinal strain, *VTI* velocity time integral measured at the left ventricular outflow tract, *TAPSE* tricuspid annular plane systolic excursion, *FAC* fractional area change, EDA end diastolic area, *FWS* free wall strain, *TV s’* tricuspid valve tissue colour doppler systolic velocity (free wall), *hsTnT* high sensitivity Troponin T, *VIS* vasopressor–inotrope score, *IMV* invasive mechanical ventilation. *P* values refer to normal vs. abnormal RV systolic function

### Association between myocardial injury and echocardiographically defined systolic dysfunction

There were no significant differences in incidence of LV or RV dysfunction between patients with and without acute myocardial injury. However, there were statistically significant differences in indices of longitudinal contractility, MAPSE and TAPSE. LV GLS and RV FWS were numerically increased (ie. impaired strain) in patients with acute myocardial injury; however, these differences were not statistically significant (Table 4).

**Table 4:** Echocardiographic variables stratified by acute myocardial injury

	No acute myocardial injury	Acute myocardial injury	*P* value
LVEF	64.5 (58 to 71)	62 (52 to 66.5)	0.078
MAPSE avg	14 (12 to 14)	11 (8 to 12)	<0.001
MV S’ avg	5.9 (5.13 to 6.76)	6 (4.85 to 7.51)	0.652
LV GLS	−21.5 (−22.6 to −16.7)	−16.5 (−20.1 to −14.2)	0.069
VTI	20.2 (16.7 to 25.6)	18.3 (15.3 to 21.4)	0.126
TAPSE	21 (19.5 to 22.5)	18 (15.5 to 21.5)	0.011
FAC	50 (44 to 53)	45 (37 to 51)	0.095
RV:LV–EDA	0.55 (0.53 to 0.59)	0.52 (0.44 to 0.57)	0.110
RV FWS	−26 (−34.7 to −21.15)	−23 (−26.1 to −20.7)	0.105
TV S’	11 (8.6 to 12)	9.4 (8 to 12)	0.518
LV dysfunction	1 (11%)	14 (32%)	0.418
RV dysfunction	0 (0%)	13 (28%)	0.055

All variables are given as median (IQR) or number (%) unless otherwise stated

*LVEF* left ventricular ejection fraction, *MV s’* mitral valve tissue colour doppler systolic velocity (average of septal and lateral measurements), *MAPSE* mitral annular plane systolic excursion, *GLS* global longitudinal strain, *VTI* velocity time integral measured at the left ventricular outflow tract, *TAPSE* tricuspid annular plane systolic excursion, *FAC* fractional area change, *EDA* end diastolic area, *FWS* free wall strain, *TV s’* tricuspid valve tissue colour doppler systolic velocity (free wall)

Peak hsTnT differed significantly between patients with and without LV dysfunction (126 (50–284) ng/l vs. 43 (16–105) ng/l, *p*=0.006) as well as for patients with and without RV dysfunction (85 (52–239) ng/l vs. 47 (16–117) ng/l, *p *= 0.026) (Tables 2 and 3). NTproBNP was significantly higher in patients with LV dysfunction compared to those without (5220 (2380–19500) vs. 1190 (630–3619) ng/l, *p *= 0.011), but not in patients with RV dysfunction (5220 (1970–12600) vs. 1720 (660–4280) ng/l, *p *= 0.062).

### Association between acute myocardial injury, echocardiographic findings and 30-day all-cause mortality

Patients who died within 30 days of ICU admission had higher SAPS-3 score and tended to be older and more frail. There were no significant differences in the majority of other baseline characteristics, and need for respiratory, vasopressor or renal replacement support. Acute myocardial injury, myocardial injury, severe myocardial injury and peak hsTnT concentrations were not associated with 30-day mortality. LV or RV dysfunction, or any or the individual echocardiographic variables were not significantly different between survivors and nonsurvivors (Additional file 1: Table S4). There were also no differences in terms of need for IMV, days spent in IMV and ICU length of stay among those with and without LV and RV dysfunction (data not shown).

### Sensitivity analyses

Sensitivity analyses were conducted using two alternative definitions of myocardial injury. When defined as hsTnT > 14 ng/l the incidence of myocardial injury was 82%. LV or RV dysfunction was not associated with this definition of myocardial injury. MAPSE and TAPSE were significantly impaired in the group with myocardial injury (Additional file 1: Table S4). The proportion with myocardial injury defined as hsTnT > 14 ng/l was not different between 30-day survivors and nonsurvivors (79% vs. 100%, *p *= 0.107, Additional file 1: Table S4).

When myocardial injury was defined as ‘severe’, i.e., hsTnT > 45 ng/l the incidence in the overall population was 57%. Using this definition there were no differences in the proportion of survivors vs. nonsurvivors with myocardial injury (53% vs. 77%, *p *= 0.106, Additional file 1: Table S5). RV dysfunction was also not associated with this this definition of myocardial injury, although LV dysfunction, MAPSE and TAPSE were significantly impaired (Additional file 1: Table S5).

## Discussion

This study demonstrates that AMInj occurs commonly in patients admitted to the intensive care unit with COVID-19, approximately 1 week after ICU admission. A majority of patients also had preexisting troponin elevations above the URL of a normal healthy population (hsTnT > 14ng/l) on ICU admission. Left and right ventricular systolic dysfunction occurred less commonly, in 28% and 22%, respectively, when defined as a composite of at least 2 echocardiographic markers. Using this conservative definition neither left nor right ventricular systolic dysfunctions were associated with AMInj, days on invasive mechanical ventilation, ICU LOS or 30-day mortality.

Acute myocardial injury, defined according to the 4th universal definition of myocardial infarction [13] occurred in 82% of the population, and 59% of patients had increased hsTnT above URL at ICU admission. Despite the high incidence of myocardial injury at admission, we found that further acute injury occurred during ICU admission, and this was in turn associated with increased time in mechanical ventilation, vasopressor and inotrope support, and longer ICU stays. Thirty-day mortality was higher among patients with AMInj (22% with vs. 0% without) but this arguably clinically significant finding did not reach statistical significance. Supporting its clinical significance are the findings of the sensitivity analyses, and that maximal SOFA scores, ICU LOS, and ICU mortality are all higher among those with AMInj. Peak hsTnT occurred about 1 week later in those with AMInj, compared to those without, indicating that while myocardial injury was common at ICU admission, patients suffer an additional, acute injury that is linked with poorer clinical outcomes. This is a novel finding, since previous studies have only defined myocardial injury as cTns above URL of a normal healthy population, and thus not able to identify the acuity of the event in a population that is likely to be exposed to preexisting cTn elevations. This unexpected observation begets the question of whether AMInjmay be related to the disease process itself, critical illness or its management. We cannot answer this question and can only suggest that cTns be monitored in this group of patients, while we seek methods of mitigating this injury.

It is not inconceivable that AMInj may occur in patients with COVID-19 given the cardiac tropism of the virus, particularly against a background of critical illness, where numerous other risk factors may exist. Clinical symptoms and ECG findings may not reliably detect myocardial injury, drawing parallels with the detection of myocarditis that is demonstrable on CMRI but not by other conventional means [14–16]. Previous studies are generally limited by selection and ascertainment biases, since cTn measurements were not routinely available for all patients and only measured if there was a clinical indication. Thus, the association between increased cTns and mortality seen in previous studies may have been a reflection of disease severity or underlying cardiovascular comorbidities rather than the occurrence of an acute myocardial injury itself, that is translated to increased mortality rates. By enrolling consecutive patients admitted to ICU, our study provides a better indication of the prevalence of myocardial injury and ventricular systolic dysfunction in the general COVID-19 ICU-population.

NTproBNP was increased in patients with and without myocardial injury confirming previous studies [26–28]. However, in our population NTproBNP concentrations were significantly higher in the group with myocardial injury (*p* < 0.001). Median peak NTproBNP was 1960 ng/l, which is surprisingly high, considering van den Heuvel et al. [27] found levels > 1000 ng/l in only 8% [26]. NTproBNP also peaked before hsTnT (day 4 vs. day 8) suggesting that myocardial stretch preceeds injury, and that the latter may be amenable to treatment.

RV dysfunction was detected in 22% of our cohort using the composite echocardiographic definition, similar to many other studies which have reported frequencies ranging from 10 to 39% [17–21], even though our cohort consisted entirely of critically ill patients with a majority requiring invasive mechanical ventilation. Similarly, the incidence of LV dysfunction was 28%. This also falls within the range of previously reported incidences in hospitalized patients [17, 18, 21]. We did not find a predominance of RV dysfunction, as reported in previous studies [18–20]. It may be argued that the incidences of left and right ventricular dysfunctions are lower than expected for this population of severely ill patients, compared to other cohorts of hospitalized patients. One possible explanation is that our definitions of RV and LV dysfunction were more stringent than in previous studies, requiring the presence of at least 2 echocardiographic criteria. Another possible explanation was that echocardiographic examinations were performed within 72 h of admission and did not capture abnormalities appearing later during ICU stay. Finally, echocardiography was conducted in all patients admitted to ICU at our institution, thus we avoided falsely elevated prevalences due to selection and indication bias.

Although left and right ventricular dysfunctions were more common in patients with AMInj, this difference did not reach statistical significance even if they may arguably be clinically meaningful (AMInj in 32% vs. 11% for LV dysfunction, 28% vs. 0% for RV dysfunction). When examining the echocardiographic variables individually, there were clinically and statistically significant differences in the indices of longitudinal function, MAPSE and TAPSE. These findings raise the question of whether these longitudinal indices of contractility may be more sensitive indicators of myocardial pathology in critically ill patients with COVID-19 disease and are in support of the systematic review of Messina et al. [21] suggesting that focal left ventricular abnormalities may be present despite a normal global LVEF. MAPSE has been demonstrated to be a principal contributor to LVEF [29] and is more sensitive in detecting LV dysfunction than EF in patients with cardiovascular disease [30, 31]. Although GLS is considered a sensitive indicator of LV systolic function, previous studies have demonstrated the superior feasibility and utility of MAPSE for predicting left ventricular strain in the critically ill [32, 33]. Since subendocardial myocardial fibres are longitudinal in orientation and most likely to be affected by oxygen demand-supply imbalances, there is a physiologic rationale to their association with myocardial injury especially within the context of hypoxaemic respiratory failure. Despite their simplicity, and not withstanding all limitations related to the unidirectional and single-level nature of these measurements, the feasibility of MAPSE and TAPSE in mechanically ventilated critically ill patients make these variables deserving of further validation.

While the current study had almost complete data on hsTnT and included consecutive ICU patients, we acknowledge its single-centre nature and the limited sample size. This study is underpowered and 30-day mortality outcomes did not reach statistical significance among patients with and without AMInj. A true difference may, therefore, have been missed and is supported by significant differences in the other clinical outcome measures and the sensitivity analyses.

Consecutive enrolment of patients was possible, since we were privileged to have echocardiography and hsTnT measurement as a standard routine at our centre, yet echocardiography was not conducted in 20 patients within 72 h of admission due to extreme clinical workloads. Thus, while we sought to minimize biases due to selection and confounding by indication that is a universal limitation with previous studies, our monocentric sample still represents only a select group of critically ill patients with COVID-19 and the findings may not be generalizable.

Other limitations include the lack of evaluation of the nature of acute myocardial injury. We did not routinely conduct 12-lead ECGs on all patients and did not make any formal evaluation of ischaemic symptoms and signs, which would have helped differentiate between acute myocardial injury and acute myocardial infarction (Types I and II). We did not measure organ perfusion pressures or tissue oxygenation. We are also unable to report upon the reversibility of these changes, since follow-up biomarker data were not universally available.

## Conclusions

Acute myocardial injury is very common in patients with COVID-19 treated in the ICU, occurring in over 80% of patients about 1 week after ICU admission and has a negative impact on clinical outcome. Right ventricular and left ventricular dysfunctions occur less commonly, affecting 22% and 28% of this population. Echocardiographic indices of longitudinal contractility were associated with acute myocardial injury but there was no relationship with clinical outcome. The association between acute myocardial injury and echocardiographic indicators of systolic function, as well as its independent association with clinical outcome requires confirmation in prospective studies.

## Supplementary Information

**Additional file 1:** **Table S1.** PRICE utility checklist. **Table S2.** Multiple linear regression analysis showing association between acute myocardial injury and ICU LOS and days on mechanical ventilation. **Table S3.** Variables during echocardiographic examination. **Table S4.** Baseline characteristics, biomarkers, myocardial injury and echocardiographic findings among survivors and non-survivors. **Table S5.** Incidence of LV and RV dysfunction in patients with myocardial injury using 2 alternative definitions of myocardial injury.

## Data Availability

The data from this study will be made available after publication, upon application to the corresponding author and within the terms of the Global Data Protection Regulation and the Swedish Patient Data Law (2008:355). To avoid the possibility of identifying individual cases, detailed data are not given in the paper but may be requested from the corresponding author.

## Abbreviations

SAPS: Simplified acute physiology score
SOFA: Sequential organ failure assessment
CRRT: Continuous renal replacement therapy
hsTnT: High sensitivity Troponin T
NT-proBNP: N-terminal pro B-type natriuretic peptide
ICU: Intensive care unit
LOS: Length of stay
IMV: Invasive mechanical ventilation
PEEP: Positive end-expiratory pressure
LVEF: Left ventricular ejection fraction
MV s’: Mitral valve tissue colour doppler systolic velocity (average of septal and lateral measurements)
MAPSE: Mitral annular plane systolic excursion
GLS: Global longitudinal strain
VTI: Velocity time integral measured at the left ventricular outflow tract
TAPSE: Tricuspid annular plane systolic excursion
FAC: Fractional area change
EDA: End diastolic area
FWS: Free wall strain
TV s’: Tricuspid valve tissue colour doppler systolic velocity (free wall)
