# Supplementary material for: Incidence of acute myocardial injury and its association with left and right ventricular systolic dysfunction in critically ill COVID-19 patients
Source: Ann Intensive Care. 2022 Jun 21;12:56. doi: 10.1186/s13613-022-01030-8 (PMC9210044; doi:10.1186/s13613-022-01030-8)
Supplement: Supplementary file 1 — Additional file 1: Table S1. PRICE utility checklist. Table S2. Multiple linear regression analysis showing association between acute myocardial injury and ICU LOS and days on mechanical ventilation. Table S3. Variables during echocardiographic examination. Table S4. Baseline characteristics, biomarkers, myocardial injury and echocardiographic findings among survivors and non-survivors. Table S5. Incidence of LV and RV dysfunction in patients with myocardial injury using 2 alternative definitions of myocardial injury. [file 13613_2022_1030_MOESM1_ESM.docx]

Table S1. PRICE utility checklist

| **PRICES CHECKLIST ITEMS** | **LV systolic function** | **RV function** |
| --- | --- | --- |
| **Research vs Clinical Study** |  |  |
| Research Study | Yes | Yes |
| Clinical Study | Yes | Yes |
| **Study Information** |  |  |
| Specific study type | Yes | Yes |
| State study design | Yes | Yes |
| Report Sample Size | Yes | Yes |
| **Patient Information** |  |  |
| Age | Yes | Yes |
| Gender | Yes | Yes |
| Height and Weight (or BMI) | Yes | Yes |
| **Comorbidities** |  |  |
| Ischaemic heart disease* | Yes | Yes |
| Atrial fibrillation* | Yes | Yes |
| Hypertension | Yes | Yes (NE) |
| HFpEF | Not provided | (NE) |
| HFrEF | Not provided | Not provided |
| Pacemaker implant present | Not provided | Not provided |
| COPD or pulmonary hypertension** | Yes (NE) | Yes |
| CKD or hemodialysis | Yes (NE) | Yes (NE) |
| **Echocardiography information** |  |  |
| Type of echo (TTE/TEE) | Yes | Yes |
| Data collected at end-expiration | No | No |
| No. of beats used for averaging | Yes | Yes |
| Vendor of ultrasound machine | Yes | Yes |
| Airway pressure trace displayed on screen | No (NE) | No (NE) |
| **Clinical information at time of echo** |  |  |
| ***Ventilation*** |  |  |
| Mode of ventilation | Yes | Yes |
| Tidal volume | Yes | Yes |
| Plateau pressure | Yes (NE) | Yes |
| PEEP | Yes (NE) | Yes |
| ***Haemodynamics*** |  |  |
| Cardiac rhythm and heart rate | Yes | Yes |
| Blood pressure | Yes | Yes |
| Inotropes, vasopressors and doses | Yes | Yes |
| **Reliability** |  |  |
| Feasibility of echo | Yes | Yes |
| Intraobserver variability | No (NE) | No |
| Interobserver variability | No (NE) | No |
| Indicate if observer blinded to treatment | Yes (NE) | Yes |
| **Statistics** |  |  |
| Sample size calculation | Yes | Yes |
| Statistician blinded to treatment | Yes | Yes |
| Address confounders if applicable | N/A (NE) | N/A |
| Internal validation, if applicable | N/A | N/A |
| **LV systolic function indices** |  |  |
| LV ejection fraction | Yes | N/A |
| Tissue Doppler S’ velocity | Yes (NE) | N/A |
| Mitral annular plane systolic excursion (MAPSE) | Yes (NE) | N/A |
| LV strain or strain rate | Yes (NE) | N/A |
| **LV size** |  |  |
| LV end-diastolic diameter/volume | No | N/A |
| **Other functional indices to aid interpretation** |  |  |
| Cardiac output | No | N/A |
| Stroke volume | Yes (VTI provided) | Yes (VTI provided) |
| Any heart valve dysfunction | No | N/A |
| **RV systolic function indices** |  |  |
| Tricuspid annular plane systolic excursion (TAPSE) | N/A | Yes |
| RV fractional area change | N/A | Yes |
| Tissue Doppler s’ velocity | N/A | Yes |
| RV strain or strain rate | N/A | Yes |
| **RV size and wall thickness** |  |  |
| RV end diastolic diameter or area | N/A | No |
| RV:LV end diastolic area ratio | N/A | Yes |
| RV wall thickness | N/A | No |
| **Other functional indices to aid interpretation** |  |  |
| PFO or other shunt(s) | N/A | No |
| Pericardial effusion | N/A | No |
| Paradoxical septal motion | N/A | No |
| Interatrial septal bowing | N/A | No |
| IVC diameter | N/A | No |

*Recorded as preexisting cardiac disease as defined in the Swedish Intensive Care Registry and defined as arrythmias, heart failure or ischaemic heart disease, or any combination of these.

** Recorded as chronic lung disease as defined in the Swedish Intensive Care Registry and defined as any chronic lung disease including asthma >3 years.

NE=Non-essential item as designated by the PRICES statement

VTI=Velocity Time Integral

Additional file 1 Table S2. Multiple linear regression analysis showing association between acute myocardial injury and ICU LOS and days on mechanical ventilation.

|  | **ICU LOS** |  |  |
| --- | --- | --- | --- |
|  | *B-coefficent* | *Std. Error* | *P-value* |
| **SAPS-3** | -0.260 | 0.201 | 0.200 |
| **SOFA on admission** | 0.569 | 0.749 | 0.450 |
| **Acute myocardial injury**  R square = 0.064 | 10.040 | 5.933 | 0.095 |
|  | **Days on mechanical ventilation** |  |  |
|  | *B-coefficent* | *Std. Error* | *P-value* |
| **SAPS-3** | -0.152 | 0.180 | 0.401 |
| **SOFA on admission** | 0.555 | 0.673 | 0.412 |
| **Acute myocardial injury**  R square = 0.086 | 11.787 | 5.325 | 0.030* |

**Additional file 1: Table S3. Variables during echocardiographic examination**.

| TV, ml/kg/PBW, median during echocardiography | 6.5(5.9-7.4) |
| --- | --- |
| Ventilator mode during echocardiography  Pressure control  Pressure support  Non invasive ventilation | 57 (79%)  3 (4%)  2 (3%) |
| PEEP, median during echocardiography, cmH_2_O, | 12 (12-14) |
| Pdrive, median during echocardiography, cmH_2_O, | 11 (9-13) |
| Ppeak, median during echocardiography, cmH_2_O, | 24 (22-27) |
| Mechanically ventilated during echocardiography | 64 (87%) |
| Vasopressors/inotropes during echocardiography | 54 (89%) |
| VIS day of echocardiography, μg/kg/min | 4.3 (1.8-7.4) |
| Sinus rhythm during echocardiography | 69 (94.5%) |
| HR during echocardiography, beats/min | 70 (60-88) |
| MAP during echocardiography, mmHg | 75 (68-85) |

All variables are given as median (IQR) or number (%) unless otherwise stated.

**Abbrevations:** TV, tidal volume; PEEP, positive end-expiratory pressure; Pdrive, driving pressure for ventilation; Ppeak, Peak pressure for ventilaton; VIS, vasopressor-inotrope score; HR, heart rate; MAP, mean arterial pressure.

VIS is calculated as dopamine dose (μg/kg/min) + dobutamine dose (μg/kg/min) + 100x epinephrine dose (μg/kg/min) + 10x milrinone dose (μg/kg/min) + 10.000x vasopressin dose (U/kg/min) + 100x norepinephrine dose (μg/kg/min)

**Additional file 1: Table S4.** **Baseline characteristics, biomarkers, myocardial injury and echocardiographic findings among survivors and non-survivors.**

|  | **Survivors**  *n* = 61 | **Nonsurvivors**  *n =* 13 | **P-value** | |
| --- | --- | --- | --- | --- |
| **Age** | 61 (55-71) | 71 (65-73) | 0.057 | |
| **Sex, male** | 42 (69%) | 8 (62%) | 0.746 | |
| **BMI, kg/m^2^** | 28.6 (25.4-31.8) | 29.4 (26.7-36.1) | 0.317 | |
| **SAPS-3** | 53 (48-60) | 64 (57-68) | 0.004 | |
| **SOFA in** | 6 (4-8) | 7 (5-9) | 0.150 | |
| **CFS** | 3 (2-3) | 3 (3-3) | 0.040 | |
| **Hypertension** | 33 (54%) | 9 (69%) | 0.317 | |
| **Diabetes mellitus** | 12 (20%) | 6 (46%) | 0.071 | |
| **Preexisting cardiac disease*** | 13 (21%) | 5 (39%) | 0.283 | |
| **Chronic respiratory disease** | 9 (15%) | 5 (39%) | 0.062 | |
| **Chronic renal disease** | 8 (13%) | 0 (100%) | 0.336 | |
| **Time from symptom onset to ICU admission, days** | 9 (7-12) | 11 (8-14) | 0.210 | |
| **hsTnT max, ng/l** | 48 (17-107) | 79 (59-141) | 0.058 | |
| **NTproBNP max, ng/l** | 1720 (740-4180) | 6550 (2540-4180) | 0.044 | |
| **Day of peak hsTnT** | 7 (3-12) | 9 (3-14) | 0.227 | |
| **Acute myocardial injury**** | 47 (78%) | 13 (100%) | 0.107 |  |
| **Severe myocardial injury***** | 32 (53%) | 10 (77%) | 0.106 | |
| **Myocardial injury****** | 48 (79%) | 13 (100%) | 0.107 | |
| **IMV days** | 14 (8-28) | 15 (4-23) | 0.390 | |
| **Vasopressor days** | 10 (5-21) | 9 (6-19) | 0.691 | |
| **CRRT** | 10 (16.4%) | 4 (30.8%) | 0.253 | |
| **ICU LOS, days** | 17 (10-32) | 15 (6-21) | 0.283 | |
| **LVEF, %** | 62 (52-67) | 63 (50-70) | 0.342 | |
| **MAPSE avg, mm** | 11 (9-13) | 10 (9-11) | 0.57 | |
| **MV S’ avg, cm/sec** | 6 (5-7) | 6 (5-6) | 0.45 | |
| **LV GLS** | -17.6 (-21.5 - -14.8) | -16.5 (-19.5 - -15.1) | 0.436 | |
| **VTI, cm** | 18.7 (15.5-21.9) | 18.5 (14.6-23.1) | 0.857 | |
| **TAPSE, mm** | 19 (17-22) | 18 (13-20) | 0.121 | |
| **FAC, %** | 47 (39-53) | 45 (38-48) | 0.326 | |
| **RV:LV-EDA** | 0.53 (0.45-0.57) | 0.54 (0.5-0.57) | 0.702 | |
| **RV FWS** | -23.3 (-28.2 - -20.7) | -23.2 (-24.8 - -22.5) | 0.738 | |
| **TV S’, cm/sec** | 9.2 (8-11.6) | 12 (6.1-13) | 0.404 | |
| **LV dysfunction** | 13 (30%) | 2 (22%) | 1.00 | |
| **RV dysfunction** | 11 (22%) | 2 (22%) | 1.00 | |

All variables are given as median (IQR) or number (%) unless otherwise stated.

**Abbrevations:** SAPS, Simplified Acute Physiology Score; SOFA, sequential organ failure assessment; CFS, Clinical Frailty Scale; ICU, intensive care unit; LOS, length of stay; CRRT, continuous renal replacement therapy; LVEF, left ventricular ejection fraction; MV s’, mitral valve tissue colour doppler systolic velocity (average of septal and lateral measurements); MAPSE, mitral annular plane systolic excursion; GLS, global longitudinal strain; VTI, velocity time integral measured at the left ventricular outflow tract; TAPSE, tricuspid annular plane systolic excursion; FAC, fractional area change; EDA, end diastolic area; FWS, free wall strain; TV s’, tricuspid valve tissue colour doppler systolic velocity (free wall). *defined as arrythmia, heart failure or ischaemic heart disease.

**defined as an increased hsTnT >14ng/L and a >20% absolute change with or without ischaemic symptoms *** defined as hsTnT >45 ng/l at any time during ICU stay; ****defined as hsTnT>14ng/l at any time during ICU stay.

**Additional file 1: Table S5. Incidence of LV and RV dysfunction in patients with myocardial injury using 2 alternative definitions of myocardial injury.**

|  | Myocardial injury(hsTnT >14ng/l) | | | Severe myocardial injury(hsTnT >45ng/l) | | |
| --- | --- | --- | --- | --- | --- | --- |
|  | No | Yes | p | No | Yes | p |
| LV dysfunction | 1 (11%) | 14 (39%) | 0.418 | 2 (9%) | 13 (42%) | 0.009 |
| RV dysfunction | 0 (0%) | 13 (28%) | 0.055 | 3 (12%) | 10 (30%) | 0.098 |
| LVEF, % | 64.5 (58-71) | 62 (52-66.5) | 0.078 | 63 (56-69) | 62 (51-67) | 0.205 |
| MAPSE, mm | 14 (12-14) | 11 (8-12) | <0.001 | 12 (11-14) | 10 (8-11) | 0.003 |
| MV s’, cm/sec | 6 (5-7) | 6 (5-8) | 0.652 | 6 (5-7) | 6 (5-10) | 0.450 |
| LV GLS | -22(-23 - -17) | -17 (-20 - -14) | 0.069 | -18(-22 - -17) | -17(-20 - -14) | 0.153 |
| VTI, cm | 20 (17-26) | 18 (15-21) | 0.126 | 20 (15-23) | 18(15-21) | 0.516 |
| TAPSE, mm | 21 (20-23) | 18 (16-21) | 0.011 | 20 (17-22) | 18 (15-20) | 0.020 |
| FAC, % | 50 (44-53) | 45 (37-51) | 0.095 | 48 (41-53) | 45 (37-53) | 0.476 |
| RV:LV EDA | 0.6 (0.5-0.6) | 0.5 (0.4-0.6) | 0.110 | 0.6 (0.5-0.6) | 0.5 (0.4-0.6) | 0.077 |
| RV FWS | -26 (-35 - -21) | -23 (-26 - -21) | 0.105 | -25 (-30 - -20) | -23 (-25 - -21) | 0.243 |
| TV s’, cm/sec | 11 (8.6-12) | 9.4 (8-12) | 0.518 | 10 (8-12) | 9.4 (8-12) | 0.921 |

All variables are given as median (IQR) or number (%) unless otherwise stated.

**Abbrevations:** LVEF, left ventricular ejection fraction; MV s’, mitral valve tissue colour doppler systolic velocity (average of septal and lateral measurements); MAPSE, mitral annular plane systolic excursion; GLS, global longitudinal strain; VTI, velocity time integral measured at the left ventricular outflow tract; TAPSE, tricuspid annular plane systolic excursion; FAC, fractional area change; EDA, end diastolic area; FWS, free wall strain; TV s’, tricuspid valve tissue colour doppler systolic velocity (free wall).
